# Vandetanib as a prospective anti-inflammatory and anti-contractile agent in asthma

**DOI:** 10.3389/fphar.2024.1345070

**Published:** 2024-05-10

**Authors:** Xiaoyue Zeng, Lu Xue, Wei Li, Ping Zhao, Weiwei Chen, Wenyi Wang, Jinhua Shen

**Affiliations:** Institute for Medical Biology and Hubei Provincial Key Laboratory for Protection and Application of Special Plants in Wuling Area of China, College of Life Sciences, South-Central Minzu University, Wuhan, China

**Keywords:** vandetanib, asthma, ion channels, abnormal contraction, inflammation

## Abstract

**Background:** Vandetanib is a small-molecule tyrosine kinase inhibitor. It exerts its therapeutic effects primarily in a range of lung cancers by inhibiting the vascular endothelial growth factor receptor 2. However, it remains unclear whether vandetanib has therapeutic benefits in other lung diseases, particularly asthma. The present study investigated the pioneering use of vandetanib in the treatment of asthma.

**Methods:** *In vivo* experiments including establishment of an asthma model, measurement of airway resistance measurement and histological analysis were used primarily to confirm the anticontractile and anti-inflammatory effects of vandetanib, while *in vitro* experiments, including measurement of muscle tension and whole-cell patch-clamp recording, were used to explore the underlying molecular mechanism.

**Results:** *In vivo* experiments in an asthmatic mouse model showed that vandetanib could significantly alleviate systemic inflammation and a range of airway pathological changes including hypersensitivity, hypersecretion and remodeling. Subsequent *in vitro* experiments showed that vandetanib was able to relax the precontracted rings of the mouse trachea via calcium mobilization which was regulated by specific ion channels including VDLCC, NSCC, NCX and K^+^ channels.

**Conclusions:** Taken together, our study demonstrated that vandetanib has both anticontractile and anti-inflammatory properties in the treatment of asthma, which also suggests the feasibility of using vandetanib in the treatment of asthma by reducing abnormal airway contraction and systemic inflammation.

## Introduction

Several tyrosine kinases, including receptor tyrosine kinase (RTK), Bruton’s tyrosine kinase (BTK) and spleen tyrosine kinase (SYK), are key mediators that play critical roles in multiple human diseases (Mocsai et al., 2010; Du and Lovly, 2018; Liang et al., 2018). For decades, tyrosine kinases have been identified as emerging therapeutic targets and inhibitors have been identified and used to treat many diseases (Wu et al., 2016; Argyropoulos and Palomba, 2018; Kim and Ko, 2020), especially lung disease (Wollin et al., 2014; Yoneda et al., 2019; Murugesan et al., 2021).

In the development of asthma, several studies have shown that tyrosine kinases play a critical role in orchestrating the systemic inflammation and other structural changes that are the defining characteristics of asthma (Wong and Leong, 2004; Wong, 2005; Guntur and Reinero, 2012). A possible mechanism might be that tyrosine kinases activate or block certain cell membrane ion channels and then regulate downstream signaling, leading to abnormal airway contraction and excessive secretion of inflammatory mediators, including chemokines, cytokines and growth factors. (Braido et al., 2005; Wong, 2005; Guntur and Reinero, 2012; Barnes, 2016). In light of these findings, the anti-inflammatory and anticontractile use of tyrosine kinase inhibitors in asthma has become an enlightened strategy. For example, treatment with a SYK inhibitor called imatinib could significantly reduce the symptom of severe airflow limitation in asthma patients (Baek et al., 2021).

Vandetanib is an oral tyrosine kinase inhibitor that has been widely used in the treatment of medullary thyroid cancer (Grande et al., 2013; Cabanillas et al., 2019; Al-Jundi et al., 2020). Other studies have demonstrated vandetanib’s potential therapeutic role in breast cancer (Spanheimer et al., 2021) and lung cancer (Morabito et al., 2010). Recently, several studies have investigated the role of vascular endothelial growth factor receptor-2 (VEGFR2) in asthma (Kim et al., 2019; Kim et al., 2020; Bolandi et al., 2021). Aberrant expression of VEGFR2 is involved in mucus hypersecretion and airway hyperresponsiveness, two common features of asthma. Therefore, we hypothesized that vandetanib, as a VEGFR2 inhibitor, is likely to have potential therapeutic effect in asthma.

The aim of the present study was to investigate the potential therapeutic properties of vandetanib in asthma. First, an asthmatic mouse model treated with vandetanib was successfully established. We found that vandetanib could significantly relieve asthmatic symptoms, including abnormal airway contraction, airway resistance, inflammatory secretions and more. Meanwhile, airway samples were isolated and analyzed. To further confirm the anti-inflammatory properties of vandetanib in airway remodelling, inflammatory mediators were detected. Further muscle tension measurements and patch-clamp recordings with specific ion channel inhibitors were applied to investigate the molecular mechanism of the relaxant efficacy of vandetanib. Compared to asthmatic mice, we found that vandetanib was able to downregulate the expressions of IL-4, IL-13, VEGFR2, VEGF and Tumor necrosis factor (TNF). Vandetanib was also able to relax precontracted mouse tracheal rings (mTRs) by altering the intercellular calcium concentration, which was regulated by certain ion channels including large-conductance Ca^2+^-activated K^+^ channels (BK channels), voltage-dependent L-type Ca^2+^ channels (VDLCCs), Na^+^/Ca^2+^ exchangers (NCXs) and nonselective cation channels (NSCCs). Together, these results clarified that vandetanib has both anticontractile and anti-inflammatory properties in asthma treatment. Our research provides an indication of the potential therapeutic value of vandetanib, which may eventually prove useful in the treatment of asthma.

## Materials and methods

### Reagents and chemicals

Vandetanib was bought from Selleckchem, Inc. (Houston, TX, United States) and dissolved in dimethyl sulfoxide (DMSO). Dexamethasone was purchased from Meilunbio (Dalian, China). SYBR Green qPCR Mix was purchased from Biosharp (Hefei, China). Acetylcholine chloride (ACh), ovalbumin (OVA) and KB-R7943 were obtained from Yuanye Bio-Technology Co., Ltd. (Shanghai, China). Bovine serum albumin (BSA), papain, collagenase H, tetraethylammonium chloride (TEA), dithiothreitol (DTT), cesium chloride (CsCl), MgATP, nifedipine, pyrazole 3 (Pyr3), paxilline (PAX), gadolinium and niflumic acid (NA) were obtained from Sigma (St. Louis, MO, United States). Phosphate-buffered saline (PBS) solution was bought from HyClone (Logan, UT, United States). Paraformaldehyde (PFA) fix solution was purchased from Servicebio (Wuhan, China). The total RNA extraction kit, cDNA synthesis kit and ribonuclease inhibitor were bought from TaKaRa (Otsu, Japan). Diethyl pyrocarbonate (DEPC)-treated H_2_O was purchased from Beyotime (Shanghai, China). TRIzol^®^ was purchased from Invitrogen (Carlsbad, CA, United States). All other chemicals haven't been mentioned were purchased from Sinopharm Chemical Reagent Co. (Shanghai, China).

### Establishment of an asthmatic mouse model

Male BALB/c mice (sexually mature) were obtained from the Hubei Provincial Center for Disease Control and Prevention (Wuhan, China). All animal experiments were designed and conducted in a specific pathogen-free (SPF)-grade laboratory as previously described with minor revision (Shi et al., 2020; Li et al., 2023; Peng et al., 2023). First, we established acute asthmatic mouse models as previously described (Li et al., 2023; Peng et al., 2023). 6-week-old mice were randomly divided into the following four groups: (1) control group, (2) asthma group, (3) vandetanib group, and 4) dexamethasone group. The asthma group, dexamethasone group and vandetanib group were exposed to OVA by injections of 3 mg/mL OVA (10 mL/kg) intraperitoneally (IP) on Days 0, 7, and 14. From Day 15, the mice were consequentially intranasal instilled with 3 mg/mL OVA (1 mL/kg, once per day). Moreover, the dexamethasone group and vandetanib group were gavaged daily with dexamethasone (1 mg/kg and 10 mg/kg, respectively) or vandetanib (12.5 mg/kg, 25 mg/kg, and 50 mg/kg). The control group was treated in parallel with PBS. Nine days after sensitization, the trachea and lungs were isolated from the euthanized mice for further experiments.

### Measurement of respiratory system resistance in asthmatic mice

Experimental mice (control group, asthma group, vandetanib group and dexamethasone group) were anesthetized by IP administration of 1% sodium pentobarbital (10 mg/kg), and tracheostomized as previously described (Shi et al., 2020; Li et al., 2023; Peng et al., 2023). The anesthetized mice were ventilated with a flexiVent system (SCIREQ, Montreal, PQ, Canada). Then the measurements of the resistance of the respiratory system (Rrs) were conducted. Aerosolized ACh at 3.125, 6.25, 12.5, 25, and 50 mg/mL concentration were gradually added and the dose-response curves of different experimental groups (control, asthma, dexamethasone and vandetanib) were charted. The Rrs results were collected and analyzed in Flexiware 8 software.

### Histological analysis

Histological experiments were conceived and carried out as previously described (Shi et al., 2020; Li et al., 2023; Peng et al., 2023). Briefly, tracheal and left lung samples were isolated from experimental groups (control, asthma, dexamethasone and vandetanib). Then the isolated specimens were fixed in 4% paraformaldehyde (PFA) for 12 h at room temperature. Standard histological protocols were employed by Servicebio (Wuhan, China) to perform routine staining experiments such as hematoxylin and eosin (H&E) staining and periodic acid-Schiff (PAS) staining. The bright-field photographs of stained sections were labeled and analyzed. The PAS-positive cells in lung were counted and analyzed by using Fuji ImageJ.

### Reverse transcription and quantitative real-time PCR

After the homogenization of the right lungs of mice, total RNA was extracted, and cDNA was synthesized. Real-time PCR was then run using SYBR Green qPCR Mix on an Applied Biosystems 7500 Fast Real-Time PCR System (Foster City, CA, United States) in standard mode as described previously (Shi et al., 2020; Li et al., 2023; Peng et al., 2023). The mRNA expressions of related genes were normalized with 2^−ΔΔCt^ method. The primers were as follows: IL-13-F, 5′- CAC​ACA​AGA​CCA​GAC​TCC​CC -3′; IL-13-R, 5′- CCA​GGG​ATG​GTC​TCT​CCT​CA -3′; IL-4-F, 5′- AAC​GAA​GAA​CAC​CAC​AGA​GAG​TG -3′; IL-4-R, 5′- CGA​TGA​ATC​CAG​GCA​TCG​AAA​AG -3′; TNF-F, 5′- TGG​AAG​ACT​CCT​CCC​AGG​TA -3′; TNF-R, 5′- ACG​GCA​TGG​ATC​TCA​AAG​AC -3′; VEGF-F, 5′- ATG​GAT​GTC​TAC​CAG​CGA​AGC​TAC​TG -3′; VEGF-R, 5′- GGT​TTG​ATC​CGC​ATG​ATC​TGC​A -3′; VEGF2-F, 5′- CACCTGCCAGGCCTGCAA -3′; VEGF2-R, 5′- GCTTGGTGCAGGCGCCTA -3′. Actin was used as an internal control in the experiment, with the primers Actin-F (5′- AGA​GGG​AAA​TCG​TGC​GTG​AC -3′) and Actin-R (5′- CAA​TAG​TGA​TGA​CCT​GGC​CGT -3′).

### Tension measurement on isolated mouse tracheal rings

As described in our previous publication (Shi et al., 2020; Li et al., 2023; Peng et al., 2023), physiological salt solution (PSS) was prepared for tension measurement. Trachea and lung tissue were removed from euthanized mice and quickly transferred to ice-cold PSS. Then 6 mm mTRs were excised and suspended in a 6 mL organ bath filled with PSS at 37°C. After a 60 min equilibration (fresh PSS was refilled every 15 min), high K^+^ (80 mM) or ACh (100 μM) was employed to evoke a successive precontraction on each mTR. Then, tension measurements were conducted. According to the experiments, vandetanib or certain ion channel inhibitors were added to the organ bath. In the study of NCX, 135 mM NaCl was replaced with 135 mM LiCl.

### Measurement of channel currents on isolated mouse airway smooth muscle cells

Mouse airway muscles were isolated into single mouse airway smooth muscle cells (mASMCs) with mASMC dissociation buffer as previous description (Li et al., 2023; Peng et al., 2023). In brief, the isolated smooth muscles were digested in digest solution I at 37°C for 21–23 min and then in digest solution II at 37°C for 4–5 min. Then dissociated tissues were rinsed and carefully resuspended with 1 mg/mL BSA to harvest single mASMCs for whole-cell recording of channel currents as described previously (Shi et al., 2020; Wen et al., 2020; Li et al., 2023; Peng et al., 2023).

For the measurement of certain channel currents, isolated mASMCs were patched and immersed in bath solution. Then the VDLCC, NSCC or BK currents were recorded as described previously (Li et al., 2023; Peng et al., 2023).

### Statistical analysis

All statistical evaluations were calculated in Origin 8.0 software (OriginLab, Northampton, MA, United States). In detail, all of the data were displayed as means ± standard errors of the means (SEM). Student’s t*-*test was used. *p* < 0.05 was considered to be significant.

## Results

### Vandetanib relieved pathological changes and airway hyperresponsiveness in asthmatic mice model

**FIGURE 1:**
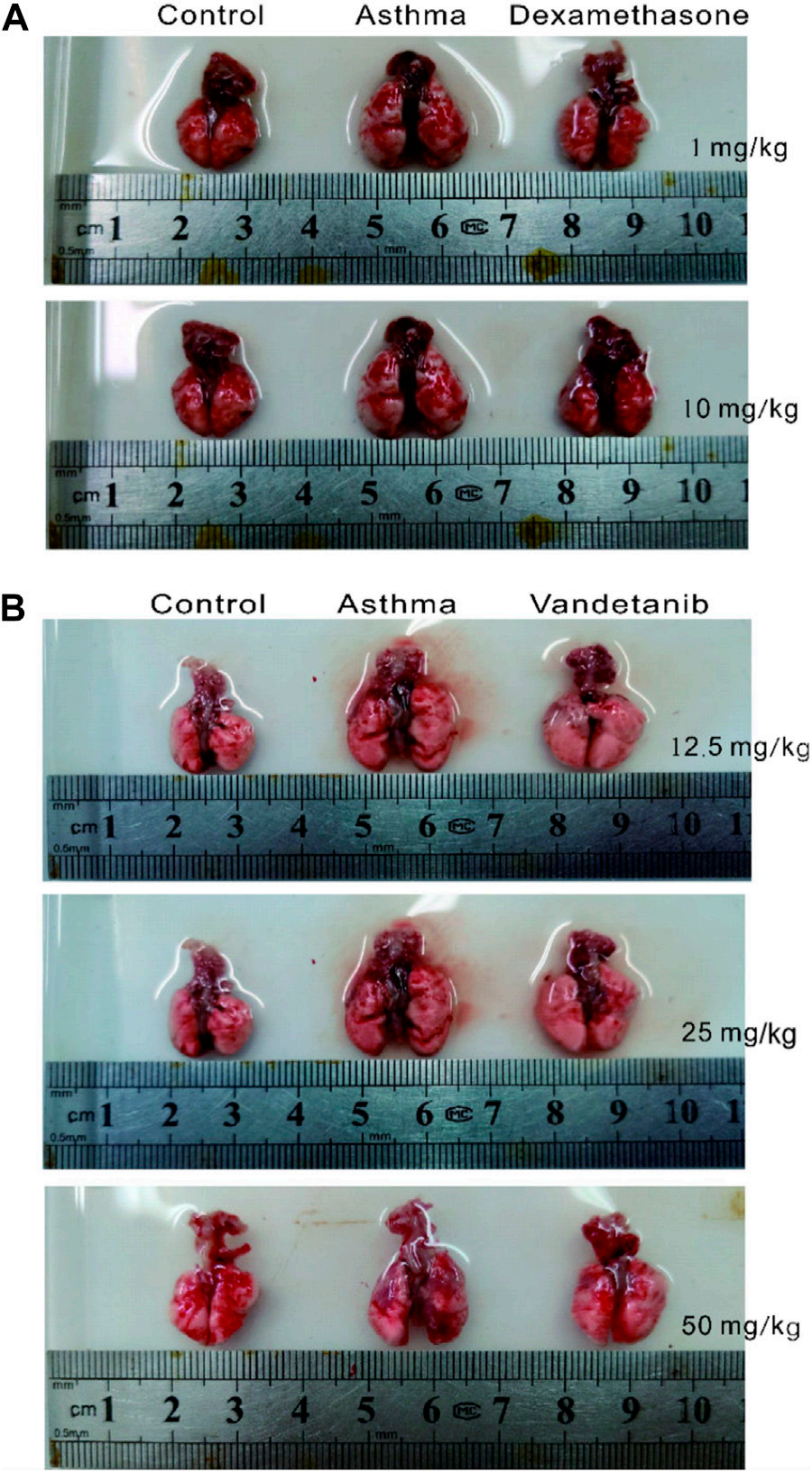
Histomorphological analysis of lungs in the control, asthma, dexamethasone and vandetanib groups. **(A)** Representative pictures of isolated lungs from the control, asthma and dexamethasone groups (1 mg/kg, 10 mg/kg). **(B)** Representative pictures of isolated lungs from the control, asthma and vandetanib groups (12.5 mg/kg, 25 mg/kg, 50 mg/kg).

In order to investigate the feasibility of vandetanib in the treatment of asthma, we established an asthmatic mouse model with vandetanib treatment (12.5 mg/kg, 25 mg/kg, 50 mg/kg). Dexamethasone treatment (Keeney et al., 2014; Wei et al., 2019) was employed as a positive control (1 mg/kg, 10 mg/kg). The trachea and lung derived from the asthma group (Figures 1A,B, middle panel) showed a series of visual changes, including enlarged trachea and lung, compared with those of the control group. To compare with the asthma group, both the dexamethasone group (Figure 1A, right panel) and the vandetanib group (Figure 1B, right panel) showed relieved enlargement of the trachea and lung. According to the results, the 12.5 mg/kg vandetanib group and 1 mg/kg dexamethasone group were chosen for further studies.

**FIGURE 2:**
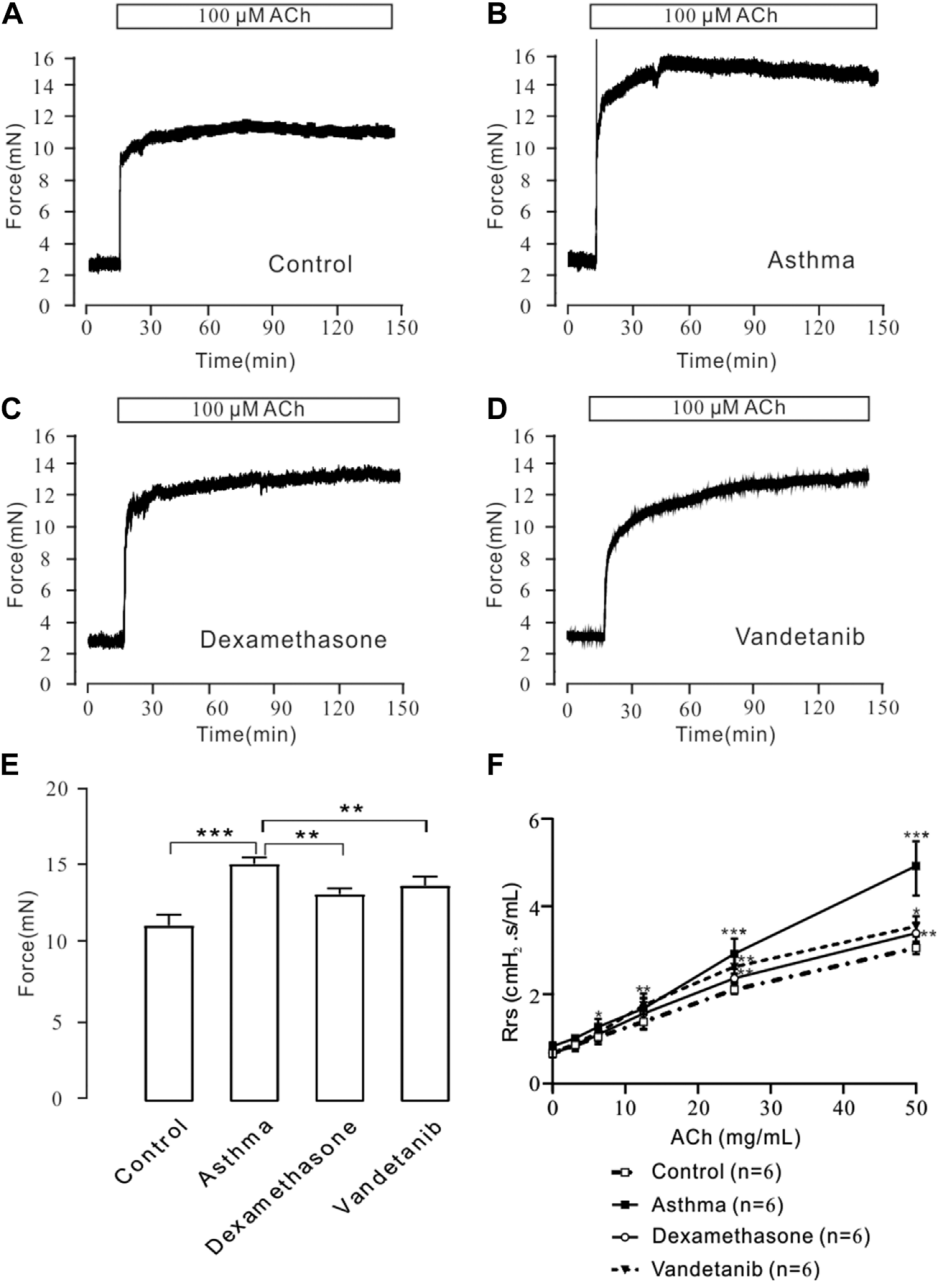
ACh-induced precontraction in asthmatic mTRs. **(A–D)** ACh (100 μM) induced stable contractions in mTRs isolated from the control, asthma, dexamethasone and vandetanib groups. **(E)** The bar graph shows the comparison of contractile forces among the control, asthma, dexamethasone and vandetanib groups (*n* = 4/4 mice). **(F)** Gradually added ACh induced an increase in Rrs in the control, asthma, dexamethasone and vandetanib groups. ACh-induced increases in Rrs were significantly inhibited in the vandetanib and dexamethasone groups compared with the asthma group (*n* = 6/6 mice). *, *p* < 0.05; **, *p* < 0.01; ***, *p* < 0.001.

To detect airway hyperresponsiveness, mTRs were were obtained from the control, asthma, vandetanib and dexamethasone groups (Figures 2A–D). ACh, which is a receptor agonist (Wang et al., 2019) was applied to induce airway contraction. The results showed that the 100 μM ACh-evoked contraction of mTRs in the asthma group (Figure 2B), was much higher than that in the control (Figure 2A), dexamethasone (Figure 2C) and vandetanib (Figure 2D) groups. Statistical data were calculated and analyzed in Figure 2E. Taken together, we successfully established asthmatic mice model. And application of vandetanib could effectively relieve airway hyperresponsiveness.

To further investigate the relaxant feature of vandetanib *in vivo*, we detected Rrs using forced oscillation technique (Figure 2F). When the aerosolized ACh was gradually administered (0, 3.125, 6.25, 12.5, 25, and 50 μg/μL), the Rrs was incrementally strengthened by ACh in a concentration-dependent way. The concentration-Rrs curve of the control group was significantly lower compared with asthma group. Moreover, the concentration-Rrs curve was significantly inhibited by 12.5 mg/kg vandetanib and 1 mg/kg dexamethasone. These results are an indication that vandetanib treatment may attenuate airway hyperresponsiveness *in vivo*.

### Vandetanib relieved airway inflammation and mucous hypersecretion *in vivo*

**FIGURE 3:**
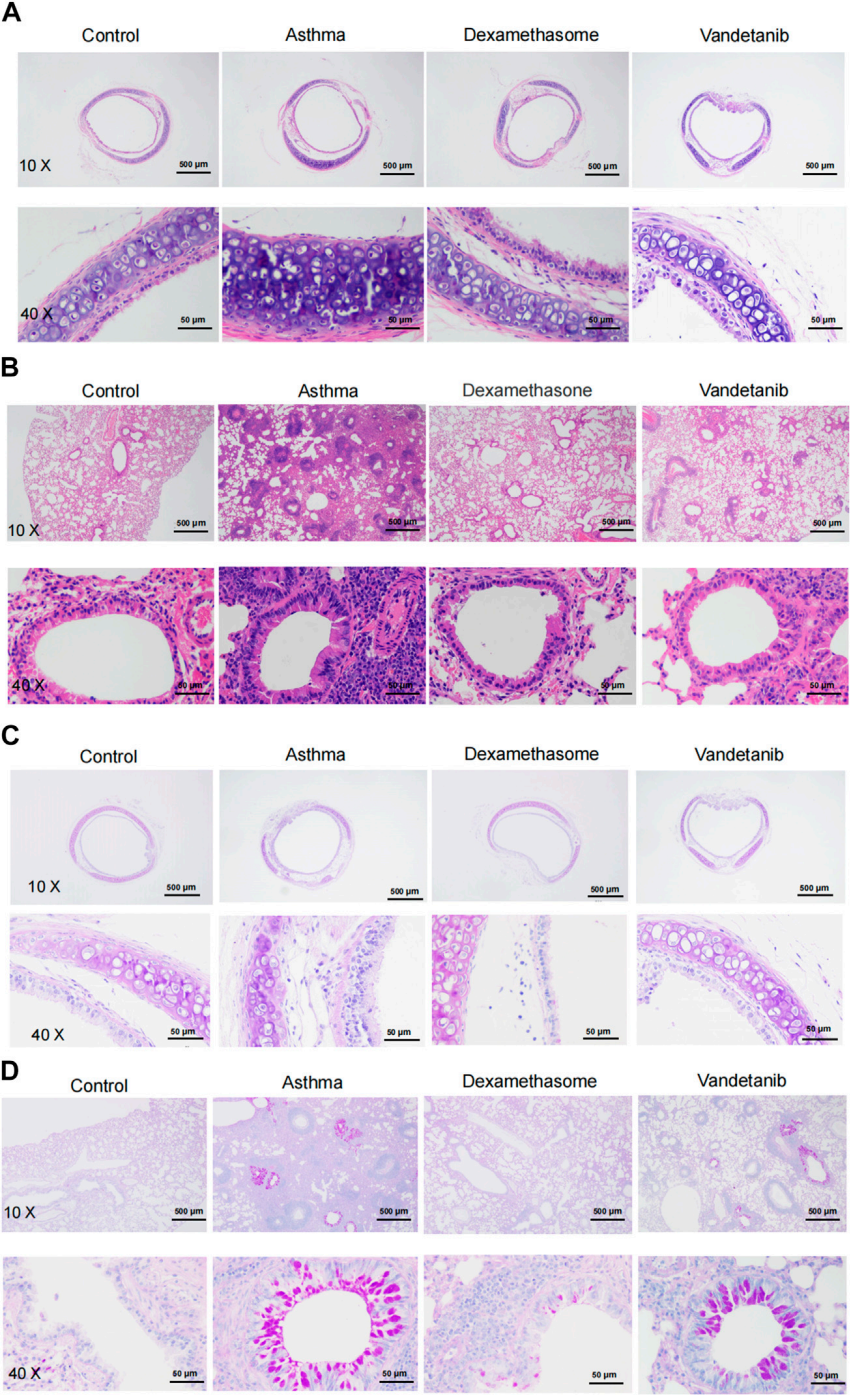
H&E and PAS staining analysis of asthmatic mouse tracheal and lung sections. **(A)** Representative tracheal images from the control, asthma, dexamethasone and vandetanib groups. **(B)** Representative lung images were obtained from the control, asthma, dexamethasone and vandetanib groups. **(C)** Representative tracheal images were obtained from the control, asthma, dexamethasone and vandetanib groups. **(D)** Representative lung images were obtained from the control, asthma, dexamethasone and vandetanib groups.

The effect of vandetanib on the asthmatic mouse model was further investigated on sliced sections of tracheal and lungs with histochemistry staining. H&E staining was applied to observe the structure of tracheal and lung specimens. In the asthma group, we observed abnormal hypertrophy of the tracheal ring with partial loss of ciliated epithelium (Figure 3A, asthma group) compared with those of the control group (Figure 3A, control group). Nevertheless, vandetanib treatment significantly reversed the thickening of the trachea and restored the loss of ciliated epithelium (Figure 3A, vandetanib group). Meanwhile, abnormal cell proliferation, infiltration of inflammatory cells and narrowing of the bronchi were observed in sections of the lungs (Figure 3B, asthma group). In contrast, inflammatory cell infiltration and bronchial narrowing were significantly reduced in the vandetanib group, suggesting that treatment with vandetanib may be able to reduce inflammation and repair damaged airways (Figure 3B, vandetanib group). Dexamethasone was used as a positive control (Figures 3A,B, dexamethasone group). Taken together, vandetanib treatment could attenuate airway structural changes *in vivo*.

PAS staining was employed to detect mucin hypersecretion in the airways. As shown in Figures 3C,D, PAS-labelled mucins were increased in lung samples from the asthma group compared to the control group. In addition, PAS-labelled mucins were significantly reduced in the vandetanib group, suggesting that vandetanib may alleviate mucus hypersecretion in the asthma group. Dexamethasone was employed as a positive control. In summary, the percentage of PAS-positive cells in the bronchus was significantly higher in the asthma group than in the control, vandetanib and dexamethasone groups (Supplementary Figure S1). Taken together, the application of vandetanib could relieve typical symptoms of asthma including airway remodeling and mucus secretion *in vivo*.

**FIGURE 4:**
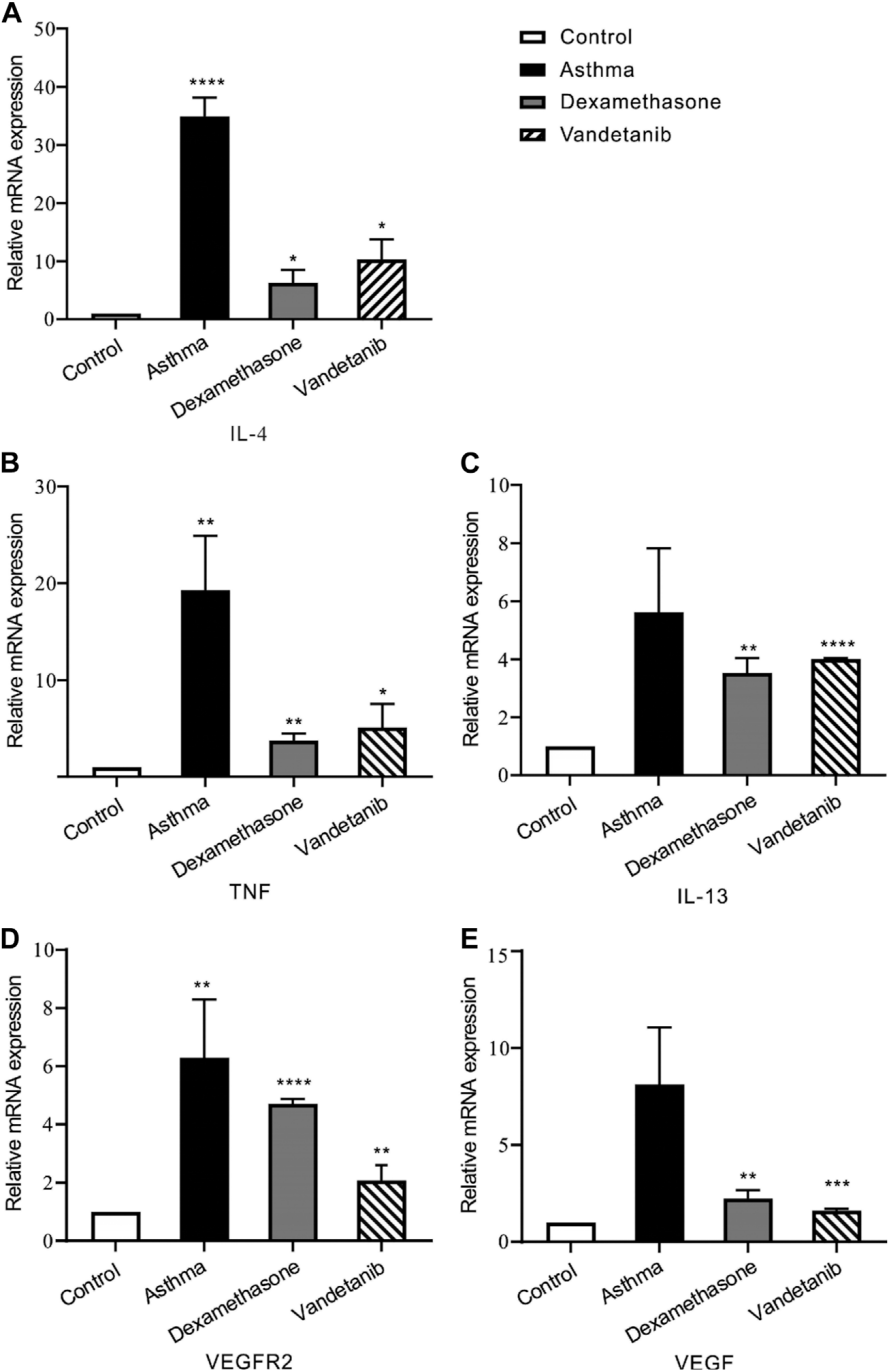
The expression levels of inflammatory cytokines and multiple tyrosine kinases in lung tissues. **(A–E)** The bar graph shows the different expression levels of IL-4, TNF, IL-13, VEGFR2 and VEGF in the control, asthma, dexamethasone and vandetanib groups. *, *p* < 0.05; **, *p* < 0.01; ***, *p* < 0.001; ****, *p* < 0.0001.

As an anti-tumor agent, vandetanib could selectively block VEGFR2 (Watanabe et al., 2021). And recent research has proven that VEGFR2 is involved in airway hypersensitivity (Bolandi et al., 2021). Given these findings, RT-PCR was applied to detect VEGF, VEGFR2, and interleukins. We found that the mRNA expression of IL-13, TNF, IL-4, VEGFR2 and VEGF, were downregulated in the control, dexamethasone and vandetanib groups compared with the asthma group (Figure 4).

### Vandetanib relaxed high K^+^-Induced precontraction in a dose-dependent manner

**FIGURE 5:**
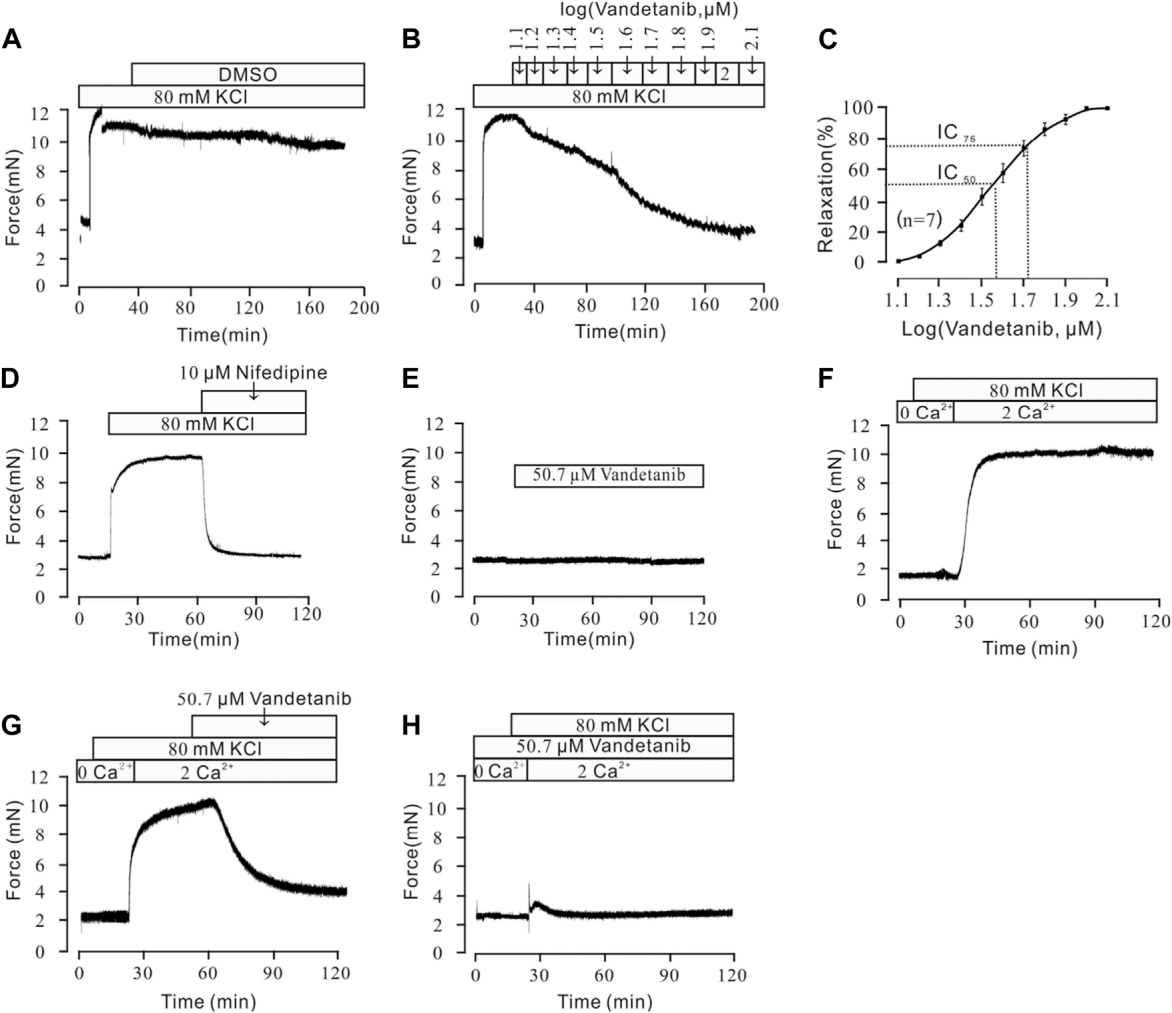
Vandetanib relaxed 80 mM K^+^-precontracted mTRs in a dose-dependent manner and blocked 80 mM K^+^-induced Ca^2+^ influx. **(A–C)** Precontraction induced by 80 mM K^+^ could be inhibited by vandetanib in a dose-dependent manner **(B)**, while DMSO could not induce relaxation **(A)**. The dose-relaxation curve is presented in **(C)** (*n* = 7/7 mice). **(D)** The 80 mM K^+^-induced precontraction was completely blocked by the VDLCC-specific blocker 10 μM nifedipine (*n* = 7/7 mice). **(E)** Treatment with 50.7 µM vandetanib had no effect on the basal tone of mTRs (*n* = 7/7 mice). **(F)** While the calcium concentration was switched from 0 to 2 mM, 80 mM K^+^-induced a steady precontraction on mTRs (*n* = 6/6 mice). **(G)** The 80 mM K^+^-induced precontraction of mTRs was almost completely erased by 50.7 µM vandetanib (*n* = 6/6 mice). **(H)** In the presence of 50.7 µM vandetanib, 80 mM K^+^ could not induce precontraction on mTRs during 0–2 mM Ca^2+^ restoration (*n* = 6/6 mice).

To further investigate the mechanistic basis of vandetanib-induced relaxation, precontracted mTRs were isolated to measure the relaxant properties of vandetanib. Our previous work showed that high K^+^ could evoke a steady precontraction of mTRs (Wen et al., 2020). Firstly, 80 mM K^+^ was applied to trigger a precontraction on mTRs. To exclude the potential effect of DMSO on precontracted mTRs, we applied DMSO to the precontracted mTRs. No relaxation was observed (Figure 5A). Compared with the results shown in Figure 5A, vandetanib gradually inhibited 80 mM K^+^-evoked contraction of mTRs in a concentration-dependent way (Figure 5B). The relevant concentration-response curve is recorded in Figure 5C. The maximal relaxation was 99.97% ± 1.8%. The half-maximal inhibitory concentration (IC_50_) was 36.24 ± 0.15 µM. The IC_75_ was 50.68 ± 0.04 µM. VDLCCs are the key ion channel during the process of high K^+^-triggered depolarization and the elevation in intracellular Ca^2+^ levels (Yaguchi and Nishizaki, 2010; Sai et al., 2014). To further explore the underlying mechanism of high K^+^-evoked precontraction, we applied a selective inhibitor of VDLCCs, nifedipine (Earl and Grivell, 2021), to the precontracted mTRs. We found that 10 µM nifedipine was able to totally relax 80 mM K^+^-induced precontraction (Figure 5D), which suggested that vandetanib-induced relaxation on mTRs might be associated with the blockage of VDLCCs. As shown in Figure 5E, no effect was observed on resting mTRs at 50.7 µM vandetanib. These results indicated that vandetanib could inhibit 80 mM K^+^-evoked contraction in a concentration-dependent way. Further usage of nifedipine implied that VDLCCs might be involved in this process.

### Vandetanib inhibited high K^+^-Induced extracellular calcium influx

Previous studies showed that intracellular and extracellular calcium mobilization participated in airway smooth muscle contraction and relaxation (Huang et al., 2020; Garriz et al., 2021). Previous study showed that VDLCCs could facilitate the influx of extracellular Ca^2+^ (Jain et al., 2020). To explore the relaxing effect of vandetanib on mTRs, muscle tension was measured to investigate the participation of Ca^2+^ in vandetanib-caused relaxation on mTRs with high K^+^-induced precontraction. It turned out that 80 mM K^+^ could not induce a contraction in 0 Ca^2+^conditions. When 2 mM Ca^2+^ was added, 80 mM K^+^ swiftly induced a constant contraction on mTRs (Figure 5F), which indicated that Ca^2+^ was necessary for 80 mM K^+^-induced precontraction. The addition of 50.7 µM vandetanib could relax 80 mM K^+^-evoked contraction under 2 mM Ca^2+^ conditions (Figure 5G). Furthermore, 80 mM K^+^ could not evoke contractions on mTRs in the presence of 50.7 µM vandetanib in Ca^2+^-free conditions or subsequent 2 mM Ca^2+^ addition (Figure 5H). These results further confirmed that VDLCC-induced extracellular calcium influx might be essential for vandetanib-induced relaxation.

### Vandetanib inhibited VDLCC currents

**FIGURE 6:**
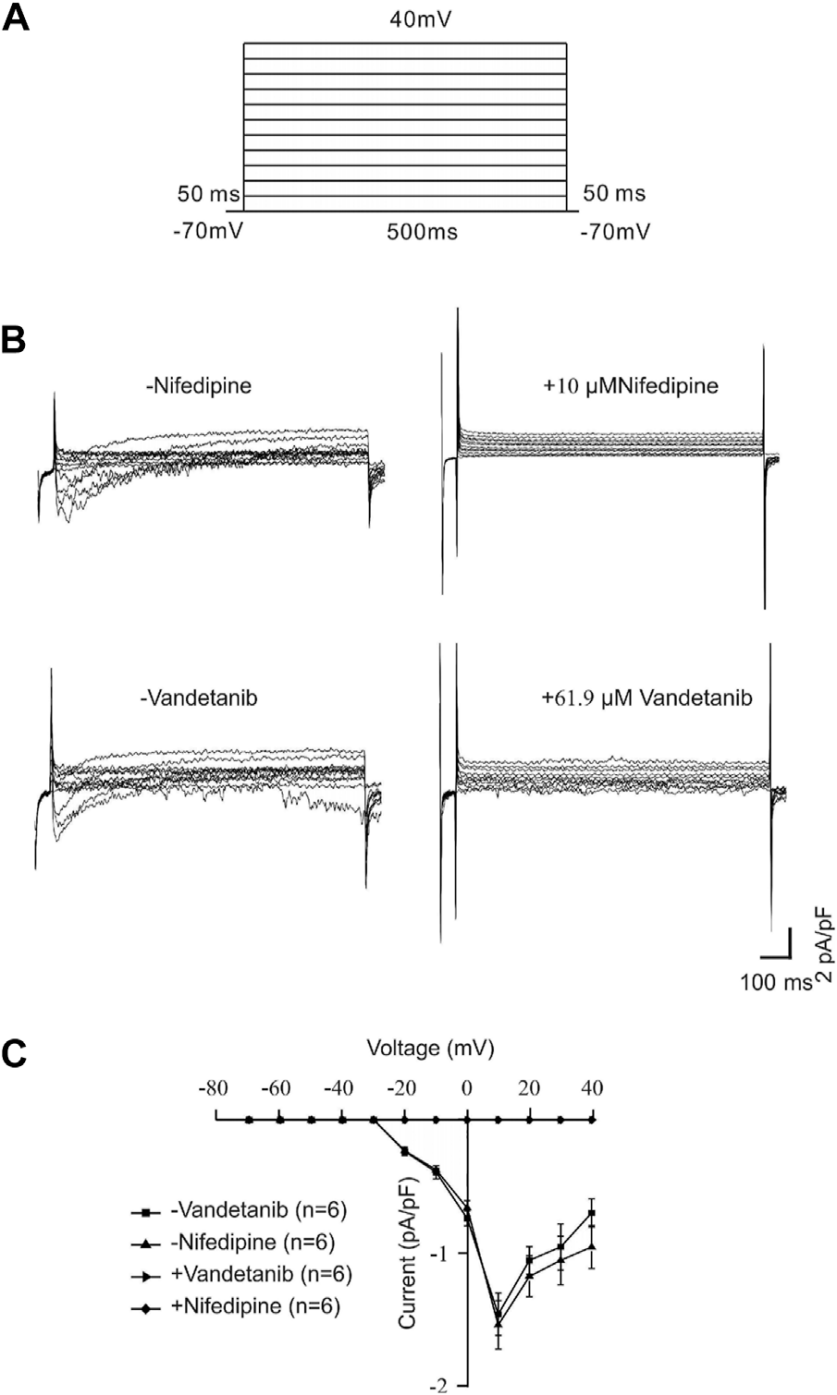
Vandetanib blocked VDLCC currents on a single mASMC. **(A)** VDLCC currents were recorded under a stepped voltage ranging from −70 to +40 mV in 10 mV increments every 50 ms. **(B,C)** VDLCC currents could be abolished by the VDLCC-specific blocker 10 μM nifedipine or 50.7 µM vandetanib. The current-voltage curve is presented in **(C)** (*n* = 6/6 mice).

For further identification of the participation of VDLCCs, we measured VDLCC currents on a single mASMC (Figure 6). First of all, VDLCC currents were recorded in the range of −70 to +40 mV (Figure 6A). Then nifedipine, which is a specific VDLCC inhibitor, was applied to confirm the recording of VDLCC currents (Figure 6B, top). VDLCC currents could also be eliminated by 50.7 µM vandetanib, which was similar to the effect of nifedipine (Figure 6B, bottom). The current-voltage (*I-V*) curves of VDLCCs in the addition of nifedipine or vandetanib was present in Figure 6C. It was turned out that vandetanib could completely erase VDLCC currents. These above results indicated that vandetanib might relieve contraction by blocking VDLCCs.

### Vandetanib relaxed ACh-Induced contraction in a concentration-dependent way

**FIGURE 7:**
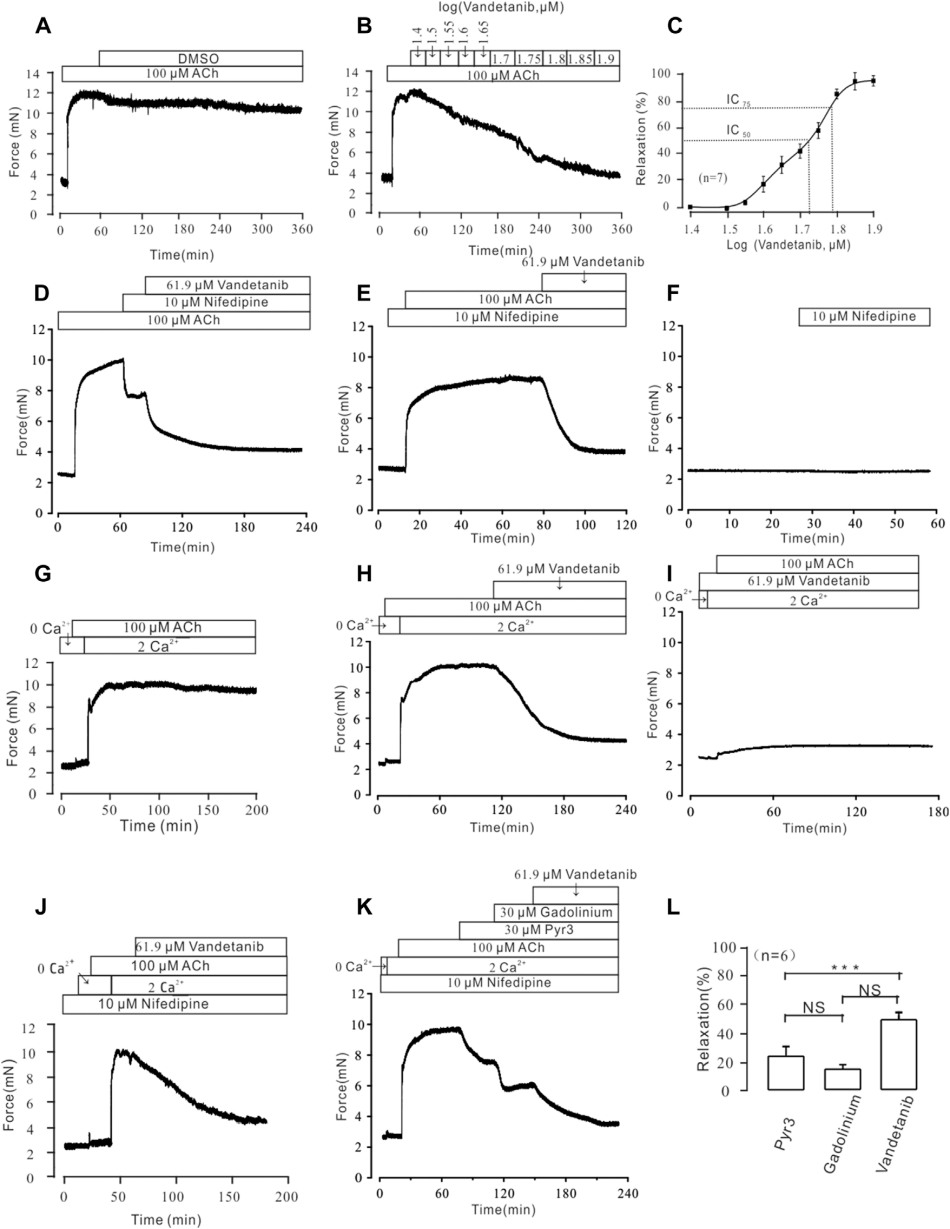
Vandetanib relaxed mTRs precontracted with 100 µM ACh in a dose-dependent manner and blocked 100 µM ACh-induced Ca^2+^ influx. **(A–C)** 100 µM ACh-induced precontraction could be inhibited by vandetanib in a dose-dependent manner **(B)**, while DMSO could not induce a relaxation **(A)**. The dose-relaxation curve is presented in **(C)** (*n* = 7/7 mice). The representative tension records are separately presented in **(A,B,D)** The 100 µM ACh-induced precontraction was partly erased by the VDLCC-specific blocker 10 μM nifedipine, and the remaining contraction was almost completely inhibited by 61.9 μM vandetanib (*n* = 6/6 mice). **(E)** In the presence of 10 μM nifedipine, ACh-induced precontraction was almost completely inhibited by 61.9 μM vandetanib (*n* = 6/6 mice). The representative tension records are separately presented in **(A,E,F)** 10 μM nifedipine has no effect on resting mTRs. **(G)** While the calcium concentration was switched from 0 to 2 mM, 100 μM ACh induced a steady precontraction on mTRs (*n* = 6/6 mice). **(H)** The 100 μM ACh-induced precontraction of mTRs was almost completely erased by 61.9 µM vandetanib (*n* = 6/6 mice). **(I)** In the presence of 61.9 µM vandetanib, 100 μM ACh could not induce precontraction on mTRs during 0–2 mM Ca^2+^ restoration (*n* = 6/6 mice). **(J)** In the presence of 10 μM nifedipine, ACh-induced precontraction was almost completely inhibited by 61.9 μM vandetanib during 0–2 mM Ca^2+^ restoration (*n* = 6/6 mice). **(K)** In the presence of 10 μM nifedipine, ACh-induced precontraction in 2 mM Ca^2+^ solution was almost completely inhibited by 30 μM Pyr3, 30 μM gadolinium, and 61.9 μM vandetanib, sequentially (*n* = 6/6 mice). **(L)** The average relaxant percentages of Pyr3, gadolinium, and vandetanib are shown in the bar graph. NS, not significant; ***, *p* < 0.001.

In addition to VDLCC, a number of other ion channels are involved in the complex development of hypertension in smooth muscle (Joseph et al., 2013). Therefore, we sought to identify ion channels other than VDLCC involved in vandetanib-induced relaxation. NSCCs and VDLCCs were involved in ACh-induced smooth muscle contraction (Wang et al., 2019). Given that, ACh was employed to precontract mTRs. First of all, we identified that DMSO solution could not relax 100 µM ACh-induced precontration (Figure 7A), which is similar to Figure 5A. Moreover, vandetanib reversed the precontraction induced by 100 μM ACh in a concentration-dependent way (Figure 7B). The IC_50_ and IC_75_ were calculated as 53.67 ± 1.72 µM and 61.9 ± 2.34 µM, respectively (Figure 7C). The maximal relaxation was 98.91% ± 1.57%. For further identification of NSCCs, nifedipine was applied to exclude VDLCCs (Figure 7D). 100 μM ACh-evoked contraction could be partly erased by 10 μM nifedipine. Then addition of 61.9 µM vandetanib eliminated the rest tension. In the presence of nifedipine, 61.9 µM vandetanib could relax 100 μM ACh-induced precontraction completely (Figure 7E). 10 μM nifedipine had no effect on resting mTRs (Figure 7F). These data identified that besides VDLCCs, NSCCs also played an important role in relaxation induced by vandetanib.

### Vandetanib blocked ACh-Evoked calcium mobilization

In addition to VDLCCs, Ca^2+^ can be transported into the cell from the extracellular solution through NSCCs (So and Kim, 2003). To investigate the involvement of calcium in vandetanib-induced relaxation, we examined extracellular calcium influx through NSCCs during ACh-evoked contraction. We found that ACh triggered a tiny and sharp contraction, which indicated that ACh released internally stored Ca^2+^ under Ca^2+^-free conditions (Figure 7G). Then the presence of 2 mM Ca^2+^ evoked a constant contraction in the presence of 100 µM ACh. Further experiments found that 100 µM ACh-evoked precontraction could be abolished by 61.9 µM vandetanib under 2 Ca^2+^ restoration (Figure 7H). However, in the addition of 61.9 µM vandetanib, ACh could not induce precontraction (Figure 7I). These results suggest that vandetanib may block VDLCCs and NSCCs, leading to the failure of ACh-induced calcium mobilization. Then the similar experiments were conducted in the addition of nifedipine (Figure 7J). In addition of 100 μM ACh, intracellularly stored Ca^2+^ was transiently released under 0 Ca^2+^ condition. The addition of 2 mM Ca^2+^ induced a constant contraction, which was abolished by 61.9 µM vandetanib. Transient receptor potential channels (TRPCs) play an important role in the release of intracellular Ca^2+^ as a major component of NSCCs (Gonzalez-Cobos and Trebak, 2010). To investigate the participation of TRPCs in vandetanib-induced relaxation, two specific blockers of TRPCs (Pyr3 and gadolinium) were applied (Wen et al., 2018; Lu et al., 2020) before the addition of vandetanib. In the presence of nifedipine, 30 µM Pyr3, 30 µM gadolinium and 61.9 µM vandetanib eliminated 100 µM ACh-evoked contraction under 2 Ca^2+^ restoration (Figure 7K). The average relaxant percentages were 23.87% ± 1.69%, 14.94% ± 2.28% and 48.73% ± 3.18% (Figure 7L). Taken together, these results suggest that vandetanib may reverse ACh-induced contraction by inhibiting NSCCs and VDLCCs. In particular, TRPCs were involved in the vandetanib-induced relaxation.

### Vandetanib inhibited NSCC currents

**FIGURE 8:**
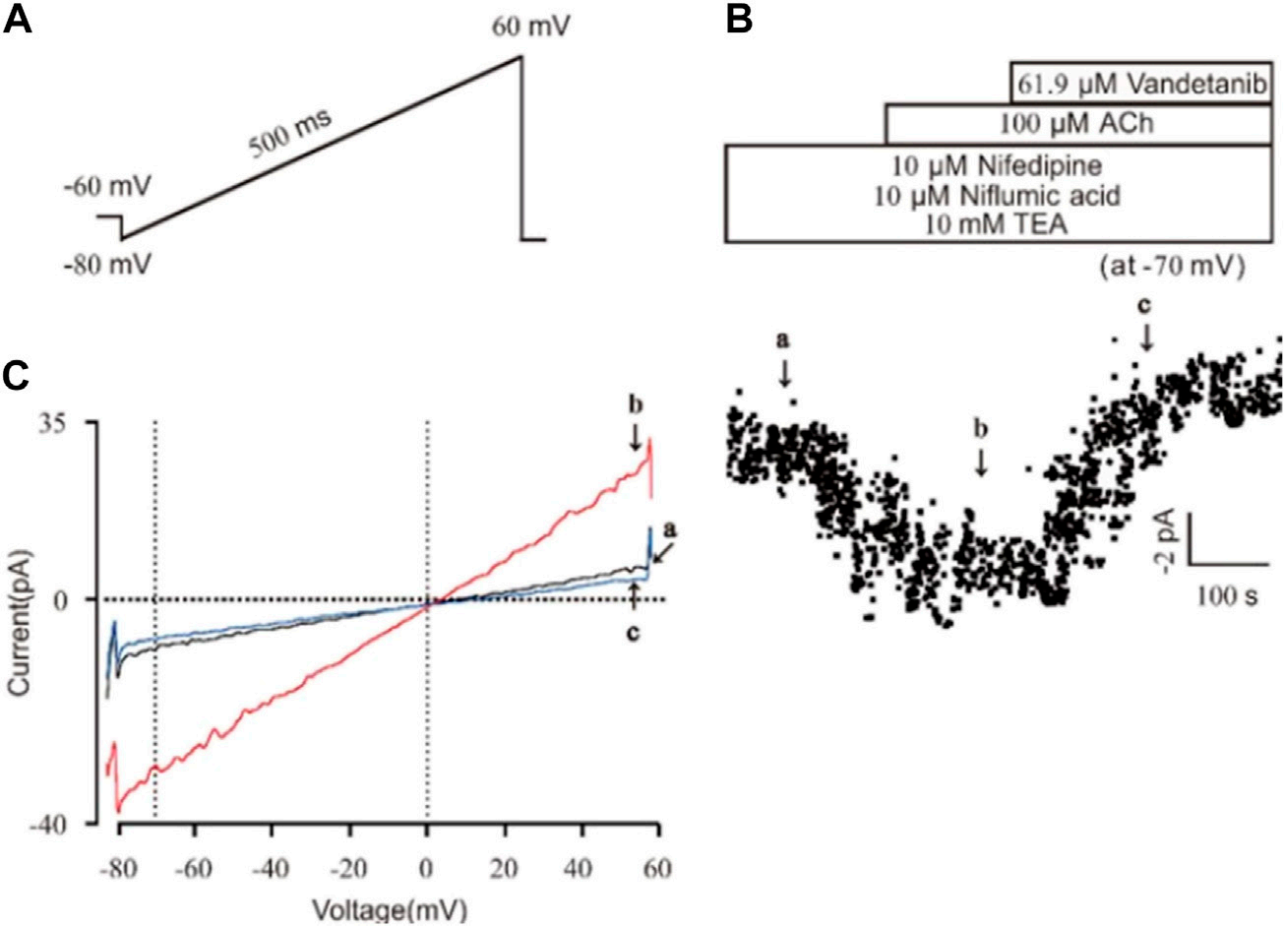
Vandetanib blocked NSCC currents on a single mASMC. **(A)** NSCC currents were recorded with a 500 ms ramp from −80 to +60 mV in 500 ms. **(B)** For isolation of NSCC currents, VDLCC currents were inhibited by 10 μM nifedipine. Cl^−^ currents were inhibited by 10 μM NA. K^+^ currents were inhibited by 10 mM TEA. Then, 100 μM ACh-induced NSCC currents were inhibited by 61.9 μM vandetanib (*n* = 6/6 mice). The data at −70 mV were used to plot current-time traces. **(C)** The net ramp currents at times a, b, and c from −80 to +60 mV.

Then we investigated the efficacy of vandetanib on NSCC currents. A ramp voltage from −80 mV to +60 mV was used to record whole-cell currents (Figure 8A). VDLCC, Cl^−^ and K^+^ currents were blocked by 10 M nifedipine, 10 M NA and 10 mM TEA, respectively, to isolate NSCC currents. The plots of the current at −70 mV are shown in Figure 8B. We found 61.9 µM vandetanib could completely inhibit NSCC currents. Figure 8C shows three representative ramp current curves at times a, b and c. This result demonstrated that besides VDLCC currents, NSCC currents could also be inhibited by 61.9 µM vandetanib.

### Vandetanib-switched NCX

**FIGURE 9:**
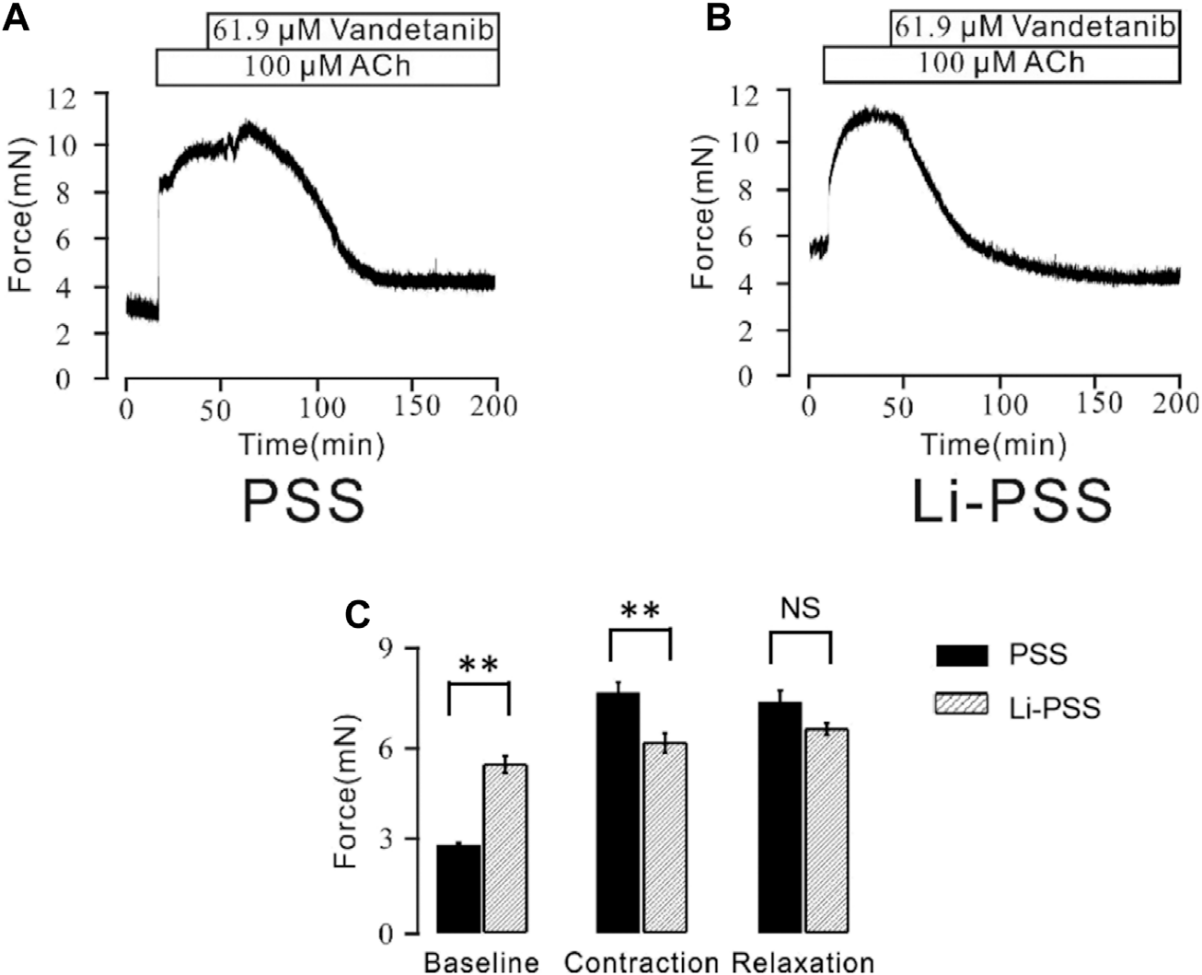
Vandetanib switched NCX. **(A)** In PSS solution, 61.9 μM vandetanib inhibited 100 μM ACh-induced precontraction (*n* = 6/6 mice). **(B)** In the Li-PSS solution, 61.9 μM vandetanib inhibited 100 μM ACh-induced precontraction. **(C)** The bar graph shows the net forces of the baseline, contraction, and relaxation (*n* = 6/6 mice). NS, not significant; **, *p* < 0.01.

Besides conventional Ca^2+^-permeable channels, VDLCCs and NSCCs, we sought to explore other specialized Ca^2+^ handling proteins that might also participate in the process of vandetanib-induced relaxation. NCX, a ubiquitous plasma membrane transporter that can drive calcium in and out by exploiting sodium, might also be a potential method for Ca^2+^ mobilization (Rose et al., 2020; Ottolia et al., 2021). To identify the involvement of NCX in vandetanib-induced relaxation, a specific inhibitor names KB-R7943 (Brustovetsky et al., 2011) was employed. As shown in Supplementary Figure S2, 5.7 μM kB-R7943 could partially 100 µM ACh-induced contraction, which preliminary indicated that NCX was also participated in vandetanib-induced relaxation. To further confirm the involvement of NCX in vandetanib-induced relaxation, Li-PSS was used to create a sodium-free condition. In addition of sodium, ACh evoked a sustained contraction (Figure 9A). Meanwhile, in the absence of sodium, the basal tone of the force was much higher (Figure 9B) compared with Figure 9A. The results suggested that NCX was switched in the absence of sodium. Then intracellular sodium was pumped out and extracellular calcium was pumped in. As a result, the intracellular Ca^2+^ increased, and the net contractile force in Li-PSS was noticeably lower than that in PSS. By adding 61.9 µM vandetanib, the contraction induced by 100 µM ACh was able to be relaxed to an even lower level than the basal tone under Li-PSS conditions. The results suggested that NCX, in the presence of vandetanib, could release intracellular Ca^2+^. Figure 9C shows the baseline, net contraction and relaxation forces. It was found that the relaxant value under PSS condition or Li-PSS condition was not significantly different. These results suggest that NCX may also be involved in vandetanib-induced relaxation.

### Vandetanib-activated K^+^ channels

**FIGURE 10:**
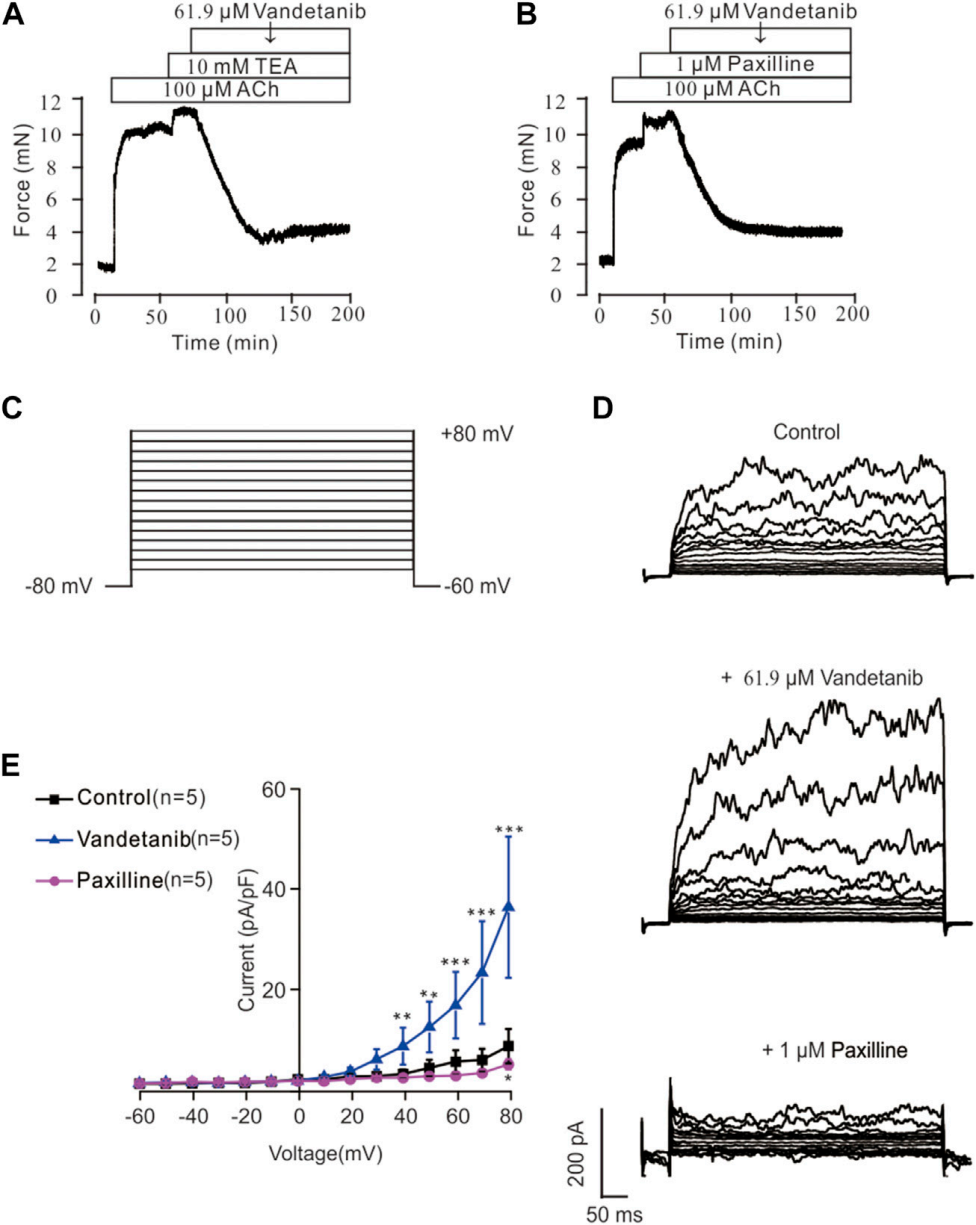
Vandetanib activated K^+^ channels and BK channels. **(A)** TEA at 10 mM significantly enhanced 100 μM ACh-evoked precontraction. The addition of 61.9 μM vandetanib almost completely relaxed the contractile mTRs (*n* = 6/6 mice). **(B)** Treatment with 1 μM paxilline significantly enhanced 100 μM ACh-evoked contraction. The addition of 61.9 μM vandetanib almost completely relaxed the contractile mTRs (*n* = 6/6 mice). **(C)** BK currents were recorded under a ramp voltage ranging from −80 to +80 mV at 10 mV increments. **(D)** Recording of K^+^ currents under the conditions of control (upper), 61.9 μM vandetanib (middle), and 1 μM paxilline (lower) from −80 to +80 mV. **(E)** A current-voltage curve was constructed based on the results of 5 cells from 5 mice. **, *p* < 0.01; ***, *p* < 0.001.

Recent studies revealed that potassium channels played a pivotal role in cellular ion homeostasis, especially Ca^2+^ (Jackson, 2018; Gao et al., 2021). In view of this, the participation of K^+^ channels in vandetanib-induced relaxation was investigated in the presence of specific K^+^ channel antagonists (Figure 10). As shown in Figure 10A, the K^+^ channel antagonist TEA (Hill and Jacques, 1999) could significantly strengthen 100 μM ACh-evoked contraction, indicating that the contraction was enhanced by K^+^ channel blockade. Subsequently, the strengthened contraction was reversed by 61.9 µM vandetanib. Paxillin, a specific inhibitor of BK channels (Duncan et al., 2021), was applied to further test the involvement of BK channels, a typical potassium channel (Rothberg, 2012), in vandetanib-induced relaxation, as shown in Figure 10B. It was found that 1 μM paxilline could enhance 100 μM ACh-evoked contraction. Addition of 61.9 µM vandetanib then reversed the contraction. Taken together, K^+^ channels, especially BK channels, might be participated in vandetanib-evoked relaxation.

### Vandetanib enhanced BK currents

BK currents were measured by patch-clamp recording at voltages ranging from −80 mV to +80 mV to further investigate the involvement of BK channels in vandetanib-evoked relaxation (Figure 10C). BK currents were detected successfully (upper) in Figure 10D. When 61.9 µM vandetanib was added, BK currents were significantly increased (Figure 10D, middle). The current was completely abolished (Figure 10D, bottom) by the addition of 1 µM paxillin. The current-voltage curve was present in Figure 10E. These results supported the hypothesis that vandetanib could enhance BK currents in mASMCs.

## Discussion

Asthma is one of the most common respiratory diseases in the world, raising public concern about increased morbidity and healthcare costs (Carr and Kraft, 2017). Current asthma medications including β-agonists and inhaled corticosteroids have limitations and severe side effects. Therefore, there is an urgent need for innovation in new drugs and therapies, as approved drugs have not been effective in reducing the symptoms of asthma (Colas et al., 2020).

Vandetanib is a tyrosine kinase inhibitor that can selectively target VEGFR2, a key mediator of airway hypersecretion and hypersensitivity. Although vandetanib has been widely used in several therapeutic areas, including NSCLC, it is not yet available for the treatment of other lung diseases, particularly asthma.

In this study, we investigated the efficacy of vandetanib in the treatment of asthma. Airway hyperresponsiveness and systemic inflammation are key symptoms of asthma. The therapeutic effects of vandetanib were primarily investigated in a mouse model of asthma. We found that mice treated with vandetanib had much less swollen and congested lungs. The ability of vandetanib to reduce airway hyperresponsiveness was then further confirmed by measurements of muscle tension and Rrs. Furthermore, histological analysis showed that vandetanib-treated asthmatic mice significantly reduced typical asthmatic symptoms including loss of ciliated epithelium, inflammatory cell infiltration, mucus hypersecretion and airway hyperresponsiveness. All of these *in vivo* results confirmed that vandetanib could alleviate key symptoms in asthmatic mice.

VEGF and VEGFR2 are validated key mediators for targeted therapy in lung disease (Pajares et al., 2012). TNF, IL-4 and IL-13 were also involved in the inflammation of the asthmatic lung (Oh et al., 2010; Lechner et al., 2021). To investigate the possible underlying mechanism, we measured the mRNA expression levels of VEGF, VEGFR2, TNF, IL-4 and IL-13. Real-time PCR results showed that the expression levels of VEGF, VEGFR2, TNF, IL-4 and IL-13 were increased in asthmatic mice, yet statistically decreased after vandetanib-treatment. The results show that vandetanib effectively reduces airway inflammation. And the possible explanation is that vandetanib inhibits the expression of VEGFR2, blocks the binding of VEGF and VEGFR2, and then further reduces the expression of downstream inflammatory factors TNF, IL-4 and IL-13.

Subsequently, the anti-contractile activity of vandetanib was evaluated in isolated mTRs. It was found that K^+^ or ACh-induced precontraction of mTRs could be significantly reversed by vandetanib in a dose-dependent manner, confirming the anti-contractile property of vandetanib. Airway muscle contraction and relaxation is a complex electrophysiological process involving calcium mobilization via ion channel regulation. The pathogenesis of asthma is activated by abnormal ion channel blockade and imbalance in calcium homeostasis. Muscle tension measurements and calcium restoration experiments with specific ion channel blockers showed that vandetanib could block VDLCC and NSCC, switch NCX and activate K^+^ channels, then relax the airways by inhibiting the influx of extracellular Ca^2+^. Further patch clamp recordings showed that vandetanib could inhibit VDLCC currents and NSCC currents, and enhance BK channel currents during the process of vandetanib-induced relaxation. *In vitro* experiments suggested that vandetanib-induced changes in VDLCCs, NSCCs, NCX and BK channels could block Ca^2+^ influx and subsequently cause airway relaxation.

Our study of vandetanib investigated the therapeutic properties of vandetanib on asthmatic mice in terms of anticontractile and anti-inflammatory activities. In summary, vandetanib could be developed as a drug candidate to alleviate asthmatic symptoms including mucus hypersecretion and airway hyperresponsiveness.

## Conclusion

*In vivo* experiments showed that vandetanib can significantly reduce a number of typical asthma symptoms, such as loss of ciliated epithelium, airway inflammation and mucus hypersecretion, by reducing receptor tyrosine kinases and inflammatory factors. *In vitro* experiments showed that vandetanib relaxes precontracted mTRs through VDLCCs, NSCCs, NCX and BK channel-regulated intercellular calcium changes. By combining the anticontractile and anti-inflammatory properties of vandetanib, our research investigated the feasibility of using vandetanib in the treatment of asthma. However, there are still some limitations to our study. The anti-inflammatory effects of vandetanib and the molecular signaling pathways involved need to be further investigated. In addition to the TRPCs, there may be other NSCCs that are related to the anticontractile properties of vandetanib, which also deserves further investigation. Further confirmation of vandetanib’s efficacy and safety as a potential inhaled drug is needed, as well as more experiments and clinical trials before it can be used in patients.

## Data Availability

The original contributions presented in the study are included in the article/Supplementary Material, further inquiries can be directed to the corresponding author.

## Supplementary material

SUPPLEMENTARY FIGURE S1 Vandetanib treatment reduced PAS-positive cells. Statistical diagram of the PAS-positive cells in control, asthma, dexamethasone and vandetanib groups respectively.

SUPPLEMENTARY FIGURE S2 NCX was involved in ACh-induced precontraction of mTRs 100 µM ACh-induced precontraction could be inhibited by 5.7 µM KB-R7943.
