# Supplementary figures and images for: Vandetanib as a prospective anti-inflammatory and anti-contractile agent in asthma
Source: Front Pharmacol. 2024 May 10;15:1345070. doi: 10.3389/fphar.2024.1345070 (PMC11116788; doi:10.3389/fphar.2024.1345070)

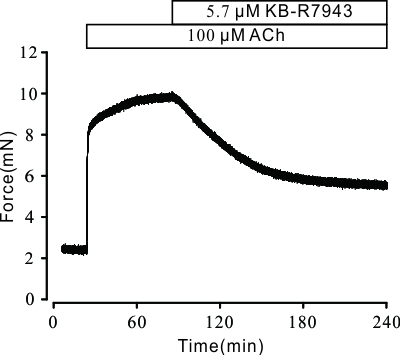

Supplement: Supplementary file 1 [file Image2.TIF]

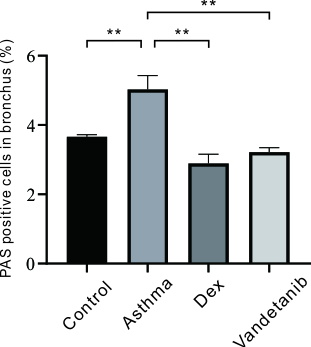

Supplement: Supplementary file 2 [file Image1.TIF]
